# Vegetation Dynamics and Food Security against the Background of Ecological Restoration in Hubei Province, China

**DOI:** 10.3390/ijerph20021225

**Published:** 2023-01-10

**Authors:** Yu Zhang, Na Gong, Huade Zhu

**Affiliations:** 1 College of Horticulture and Forestry Sciences/Hubei Engineering Technology Research Center for Forestry Information, Huazhong Agricultural University, Wuhan 430070, China; 2 The Research Center for Transformation and Development of Resource-Depleted Cities, Hubei Normal University, Huangshi 435002, China; 3 Chongqing Youth Vocational & Technical College, Chongqing 400712, China; 4 College of Urban and Environmental Sciences, Hubei Normal University, Huangshi 435002, China

**Keywords:** ecological restoration, returning farmland to forests project, vegetation dynamics, food security, Hubei Province

## Abstract

A series of ecological restoration projects have been proposed to solve ecological problems resulting from human activities. The project of returning farmlands to forests, initiated in 1999, was the most widely implemented ecological restoration project in China. Large amounts of cropland with steep slopes have been converted to forests or grasslands to promote vegetation restoration, reduce soil erosion, and control nonpoint source pollution. Therefore, identifying the dynamics of vegetation and food security is crucial for further decision making. Based on the mean normalized difference vegetation index (NDVI) and grain yield data, this study explored the vegetation dynamics and food security of Hubei Province against the background of ecological restoration. The results show that, on a whole, the NDVI significantly increased from 2000 to 2018. The spatial agglomeration of the NDVI decreased between 2000 and 2008 and then increased from 2009 onwards. High–high NDVI agglomerations were more concentrated in mountainous areas. Food security was not threatened, and the grain yield in Hubei Province and most of the cities exhibited significant upward trends, as a whole. The change trend of the grain yield was not stable during the period from 2000 to 2018. The grain yield for Hubei Province and almost all of the cities decreased during the first 5 to 11 years, probably due to the sharp decrease in the sloping cropland areas against the background of ecological restoration. Grain yield was more sensitive and had a longer downward trend in regions with steeper slopes. Increasing trends in grain yield were detected during the last 6 to 10 years for most of the cities, and this can mainly be attributed to the newly added croplands that were created from land with other kinds of land uses, the increase in grain productivity, and strict cropland protection policies. The project of returning farmlands to forests is suggested as a long-term policy from the perspective of ecological restoration, and effective measures should also be continuously taken to maintain grain production and food security.

## 1. Introduction

The economy developed rapidly at the expense of the environment in the late 20th century, which in turn caused some ecological problems, such as soil erosion, nonpoint source pollution, and land degradation. In this context, many kinds of ecological restoration programs have been initiated globally in order to rehabilitate degraded ecosystems, combat desertification, recover biodiversity, and provide ecosystem services [1,2,3], including the African Forest Landscape Restoration Initiative [4] and the Atlantic Forest Restoration Pact [5]. A series of ecological restoration projects have also been proposed in China to accelerate the restoration process of vegetation, mitigate soil erosion, and control nonpoint source pollution. The project of returning farmlands to forests, initiated in 1999, was the most widely implemented ecological restoration project in China [6]. Three kinds of land use transitions were addressed by the project of returning farmlands to forests, including changing croplands to forests, changing croplands to grasslands, and changing wastelands to forests [7]. Since then, croplands with steep slopes, especially those above 25°, have been identified for mandatory conversion to grasslands or forests. The sharp decrease in cropland areas has become a great threat to grain production [8]. However, the effect on grain production of the project of returning farmlands to forests has not been well-documented. Because achieving a balance between grain production and ecological restoration is an important issue for China, it is imperative to assess the dynamics of vegetation and food security since the implementation of the project of returning farmlands to forests in order to develop further policies for land-use management and ecological restoration.

Satellite-based imagery has been proven to provide the most significant data for research into vegetation dynamics on a large scale [9,10,11,12,13]. The NDVI is an efficient index that reflects vegetation variability, which is calculated using the red and near-infrared spectrum. Many previous studies have applied the time-series product of the NDVI to explore the vegetation dynamics in some regions where the policy of returning farmlands to forests has been implemented [14,15,16]. Most of these studies focused on the overall change trend [17] or the increase or decrease trend [18,19,20] of the vegetation, while limited attempts have been made to explore the agglomeration characteristics and the spatial and geographical dynamics of the vegetation.

Since the project of returning farmland to forests was implemented, large areas of cropland have been replaced by vegetation, which may have affected food security to some degree. Cropland resources provide the foundation for grain production, which is of great significance for maintaining food security and the sustainable development of society [21]. The conflicts between the population and cropland resources have become more prominent with population growth and economic development [22]. When cropland areas were relatively sufficient after 1960, grain yield was affected by natural factors, including climate [23], crop-rotation systems [24], and various external factors, including technological progress [25], soil fertility, and irrigation conditions [26]. Cropland areas became the main constraint for grain yield at the beginning of the 21st century, when the marginal benefits provided by external factors began to decrease [27]. Due to the implementation of the project of returning farmlands to forests, the continuous decrease in cropland areas caused a decrease in grain yield in China. However, grain yield increased after the strict farmland-protection policy was implemented in 2003 [27]. Some researchers have analyzed the spatiotemporal dynamics of grain production [28] and the effects of the driving variables on grain production [29]. However, the change trend of grain yield after implementing the project of returning farmlands to forests and the project’s effects on food security require further analysis in order to assess the conflict between grain production and ecological restoration practices.

In this study, we explore vegetation dynamics and food security and assess the effects of the project of returning farmlands to forests in Hubei Province. Hubei Province is a major grain production area in China due to its favorable climate conditions, and it plays a significant role in grain production for the country [30]. Against the background of the project of returning farmlands to forests, together with rapid urbanization, large areas of cropland were transformed to forests, grassland, and construction land, posing many challenges for grain production. The goals of this research were (1) to explore the spatiotemporal dynamics of vegetation in Hubei Province from 2000 to 2018, (2) to quantify the characteristics of change trends in grain yield and major farm crop outputs, and (3) to evaluate the effects of the project of returning farmlands to forests on vegetation restoration and grain production. The results of this study could provide us with a deeper understanding of the dynamics of vegetation coverage and food security against the background of ecological restoration and help land managers to establish more reasonable policies to achieve a balance between ecological restoration and food security.

## 2. Materials and Methods

### 2.1. Study Area

**Figure 1:**
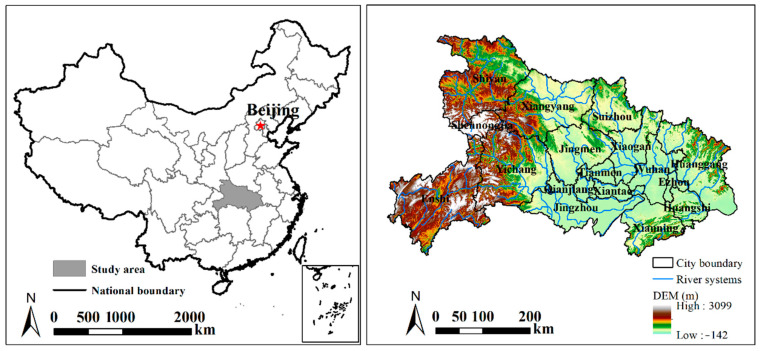
Location of Hubei Province.

Hubei Province is located at 108°21′–116°07′ E and 29°01′–33°06′ N in central China (Figure 1). It has a total area of approximately 185,900 km^2^. The ratios of mountains and hills are 56% and 24%, respectively, and they are mainly located in the west, east, and north of Hubei; the other 20% of the area comprises plains and lakes [31]. There is a big difference in the terrain from west to east. The Jianghan Plain lies in the south-central part of the province with an average elevation of approximately 27 m, and the highest elevation is 3105 m, which is located at the summit of Shennongjia. It has an average annual temperature of 15–17 °C and an average annual precipitation of 1100–1300 mm [32]. Hubei Province plays a significant role in national development strategies, including the Yangtze River Economic Belt, the Rise of Central China Plan, and the Belt and Road Initiative [33]. The GDP of the study area ranked 7th in the whole country at 3.94 trillion yuan in 2018 [34]. The total population increased from 59.60 million to 61.73 million during 2000–2018 [35,36]. This study focuses on the spatiotemporal variation of cropland and the change trend of grain yield; the results could provide a scientific reference for regions that face the same problems.

### 2.2. Data Source

The datasets used in this research included land use/land cover, topography, grain yield, and NDVI data. The land use/land cover data were acquired from the Data Centre of Resources and Environment, Chinese Academy of Science (CAS), had a resolution of 30 m, and were extracted from Landsat images. The land use/land cover contained 6 categories: cropland, forest, grassland, water, construction land, and unused land. The topography data of ASTER GDEM was provided by Geospatial Data Cloud (http://www.gscloud.cn/, accessed on 1 July 2020) and had a spatial resolution of 30 m. The city-level grain production data for every year between 2000 and 2018 were obtained from the Hubei Statistical Year Book and consisted of the three major grains, including rice, wheat, and corn. Tubers and soybean are also important grains in China, so the outputs of these two kinds of farm crops were used in the statistics and analysis of grain production in this study. The grain production data included in this study differ from the statistics of the Food and Agriculture Organization (FAO) according to China’s statistical caliber [37]. The whole province and all its cities and districts were involved in our analysis. The NDVI dataset from MODIS MOD13Q1 (version 6) during 2000 to 2018, which had a spatial resolution of 250 m and a temporal resolution of 16 days, were used to examine the variation and spatial agglomeration of the vegetation coverage. The maximum NDVI of each year was achieved using the maximum value composite (MVC) method.

### 2.3. Methodology

#### 2.3.1. Spatial Autocorrelation

Spatial autocorrelation was first presented by Anselin [38] to reflect the self-correlation of a spatial variable. The global Moran’s I and local Moran’s I are two widely applied indicators of spatial autocorrelation. These indicators were utilized to explore the characteristics of spatial distribution and the change trend of the vegetation and to identify the interaction of the composite NDVI values with the neighboring pixels. 

The global Moran’s I could be used to reflect the spatial autocorrelation of the whole study area. The formula is as follows:

(1) $$I=\frac{n}{S}\times \frac{\sum_{i=1}^{n}\sum_{j=1}^{n}w_{ij}\left(x_{i}-\bar{x}\right)\left(x_{j}-\bar{x}\right)}{\sum_{i=1}^{n}{\left(x_{i}-\bar{x}\right)}^{2}}\;\left(i,j=1,2,3,\ldots ,n\right)$$

where n refers the number of the NDVI pixels, $\bar{x}$ is the mean value of the pixels, $x_{i}-\bar{x}$ and $x_{j}-\bar{x}$ are the deviations of the pixel *i* and pixel *j* from the mean value, respectively, $w_{ij}$ is the spatial weight and represents the inverse of the distance, and *S* is the sum of all weights.

An indicator of the Moran’s I with a value above or below 0 indicates a positive or negative spatial autocorrelation, respectively. Larger values of the indicator closer to 1 mean a higher degree of aggregation for the NDVI pixels, while values closer to −1 represent larger differences between the NDVI pixels and lower concentrations of vegetation coverage [39]. If the global Moran’s I is 0, this indicates the complete randomness of the spatial pixels [40]. The significance the global Moran’s I is examined based on *Z(I)*, which is given by:

(2) $$Z\left(I\right)=\frac{I-E\left(I\right)}{\sqrt{Var\left(I\right)}}$$

where *E(I)* is the theoretical expectation and *Var(I)* is the variance of Moran’s I.

The indicator of the local Moran’s I shows the relationship between a unit and its surrounding units [38]. The significance level of the spatial association type and the spatial autocorrelation degree can be reflected by the local indicators of spatial association (LISA) [38]. The local Moran’s I can be calculated using the following formula:

(3) $$I_{i}=\frac{n\left(x_{i}-\bar{x}\right)\sum_{j=1}^{n}w_{ij}\left(x_{j}-\bar{x}\right)}{\sum_{i=1}^{n}{\left(x_{i}-\bar{x}\right)}^{2}}\;\left(i,j=1,2,3,\ldots ,n\right)$$

The variables in Equation (3) have the same meaning as those in Equation (1). A high positive local Moran’s I value suggests that the unit has a similar value to its neighbors; on the other hand, a high negative local Moran’s I shows that it is a potential spatial outlier, and dissimilar values are agglomerated [22]. The standardized statistics of the local Moran’s I were tested using the same method as that of the global Moran’s I. Four spatial clusters could be identified, including high–high, low–low, high–low, and low–high clusters. High–high and low–low clusters represent high positive local Moran’s I values, indicating high values with high neighboring values or low values with low neighboring values, respectively. High–low and low–high clusters represent high negative local Moran’s I values, indicating high and low values with low and high neighboring values, respectively [41,42].

#### 2.3.2. Mann–Kendall Trend Test

The trend analysis of the grain yield and the five specific farm crop outputs was tested by the Mann–Kendall method, which is a nonparametric, rank-based test used to assess the change trends of time-series data [43]. Its use of nonparametric techniques makes it more resilient to outliers compared to other statistical methods [17]. Moreover, the series of the data does not have to be subject to a certain distribution type. The time series of the output data is arranged sequentially. The data’s magnitude for year $x_{i}\left(i=1,\;2,\;3,\;\cdots ,\;n\right)$ is compared with that of the preceding year $x_{j}\left(j=1,\;2,\;3,\;\cdots ,\;i\right)$. The test statistic *S* is calculated via the following formula:

(4) $$S=\sum_{i=1}^{n-1}\sum_{j=i+1}^{n}sgn\left(x_{j}-x_{i}\right)$$

the *sgn* is the count of difference between *x_j_* and *x_i_* from the time series.

(5) $$sgn\left(x_{j}-x_{i}\right)=\{\begin{array}{c} \;1,\;x_{j}>x_{i}\\ \;0,\;\;x_{j}=x_{i}\;\\ -1,\;x_{j}<x_{i} \end{array}$$

The statistic *S* is approximately normally distributed when *n* ≥ 8 (*n* is the series number). In this case, the mean of the series data is 0, and the modified variance [44] is as follows:

(6) $$\mathrm{Var}\left(S\right)=\frac{n\left(n-1\right)\left(2n+5\right)-\sum_{i=1}^{n}t_{i}\left(i-1\right)\left(2i+5\right)}{18}$$

where *t_i_* refers the extent of any given time, *i* refers the number of the tied group, and *n* refers the actual series number.

The test statistic *Z* follows a standard normal distribution, and its formula is as follows:

(7) $$Z=\{\begin{array}{c} \;\frac{S-1}{\sqrt{Var\left(S\right)}},\;S>0\\ \;0,\;S=0\\ \;\frac{S+1}{\sqrt{Var\left(S\right)}},\;S<0 \end{array}\;$$

The change trend can be identified through the *Z* value. A positive value shows an upward trend, and a negative value shows a downward trend. If *Z* has an absolute value above *Z*_1−*α*/2_, this indicates that the null hypothesis of no trend is rejected [45], where the value of *Z*_1−*α*/2_ is achieved based on the standard normal cumulative distribution table. Three levels of significance were tested in this research: 0.05, 0.01, and 0.001.

The Sen’s Slope (β) is calculated as:

(8) $$\beta =Median\left(\frac{x_{j}-x_{i}}{j-i}\right)$$

where *β* represents the median value of the record pairs of the data series. If *β* has a positive value, this indicates an increasing trend, and a negative value indicates a decreasing trend.

#### 2.3.3. Sequential Mann–Kendall Test

In this study, the potential change point of the total grain yield from 2000 to 2018 was identified based on the sequential Mann–Kendall test [46,47]. The sequential values were calculated with two statistical measures, including a forward and a backward sequence [48]. The forward and the backward sequential statistics were calculated based on the original and the reverse order of the data series, respectively. These two sequential statistics constitute two curves, and a potential turning point appears when the curves of the two statistics intersect [49]. The significance is tested at the 95% level (α < 0.05). The turning point is a drastic or abrupt change in the data signaling a jump from one stable status to another stable status [50]. The magnitude of the data for each time series $x_{i}$ is compared with that of each preceding time series$\;x_{j}$. A rank sequence (*S_k_*) for time series is constructed:

(9) $$s_{k}=\sum_{i=1}^{k}r_{i}\left(k=2,3,\ldots ,\;n\right)$$

where *k* is the sequence of the year, and *r_i_* is:

(10) $$r_{i}=\{\begin{array}{c} 1,\;x_{i}<x_{j}\\ 0,\;x_{i}\geq x_{j}\; \end{array}\left(j=1,\;2,\;\ldots ,\;i\right)$$

The progressive variable statistic *UF_k_* (forward sequence) is calculated under the assumption of random and independent time series, and the formula of *UF_k_* is as follows:

(11) $$UF_{k}=\frac{\left[s_{k}-E\left(s_{k}\right)\right]}{\sqrt{Var(s_{k}})}\;\left(k=1,\;2,\;3,\;\ldots ,\;n\right)$$

where *E(s_k_)* denotes the mean of *S_k_*, and *Var(s_k_)* denotes the variance of *S_k_*. The formulas are as follows:

(12) $$E(s_{k})=\frac{n\left(n+1\right)}{4}$$

(13) $$Var\left(s_{k}\right)=\frac{n\left(n-1\right)\left(2n+5\right)}{72}$$

The retrogressive variable statistic *UB_k_* (backward sequence) is calculated with the same formula as *UF_k_* from the end of the series. Positive and negative values of *UF_k_* indicate upward and downward trends, respectively. The significance is examined at the 95% level (α < 0.05 UF_0.05_ = 1.96). A significant trend of the data series is observed during the period when |*UF*_*k*_| > *UF*_0.05_. The *UF_k_* and *UB_k_* values form the *UF* curve and *UB* curve. If these two curves intersect, the year of the intersection indicates the beginning of the change point during this period [51,52].

## 3. Results

### 3.1. Land Use Dynamics

**Figure 2:**
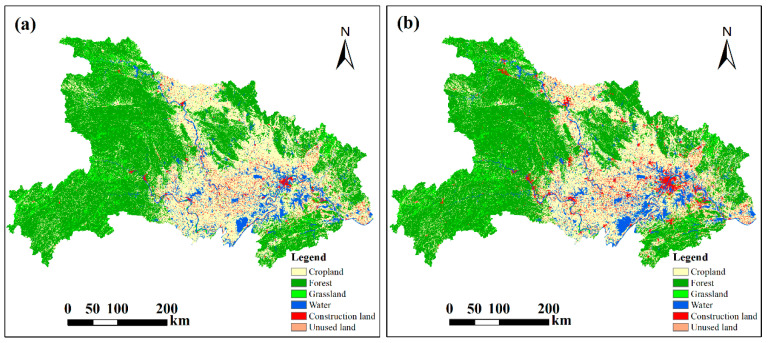
Land use of Hubei Province in 2000 (**a**) and 2018 (**b**).

The amounts and patterns of land use types changed dramatically between 2000 and 2018 (Figure 2 and Table 1). Most of the cropland was located in the Jianghan Plain, which has flat terrain. The forest land was mainly located in the mountainous and hilly areas in the west and east of Hubei Province. The major land use changes took place in cropland, construction land, and water. More cropland was distributed in the eastern than in the western parts of the study area, and the gravity center of cropland moved to the northeast during this period [30]. The area of cropland decreased from 69,650.07 km^2^ to 65,355.20 km^2^ during 2000–2018, showing a decrease of 4294.87 km^2^. Construction land area significantly increased, with an increase ratio of 67.20%. Another significant increase occurred in the water area, which increased from 10,809.80 km^2^ to 12,398.25 km^2^ during 2000–2018. In contrast, the total areas of forest and grassland remained relatively stable, and their change ratios were −0.56% and −2.28%, respectively.

Land use changes between 2000 and 2018 (Table 2) show that the decrease in cropland primarily resulted from its transition to forest and grassland. The area of cropland that was converted into forest and grassland was 4676.43 km^2^ and 285.61 km^2^, respectively, accounting for 45.68% of the total cropland decrease. In addition, substantial conversions from cropland to water and construction land were detected with areas of 2381.35 km^2^ and 3480.68 km^2^, which accounted for 21.92% and 32.04% of the total cropland decrease, respectively. Although the total amount of cropland decreased during 2000–2018, there was also newly-added cropland transitioned from other kinds of land use, which alleviated the decrease in cropland. The most remarkable cropland increase was due to conversion from forest, with an area of 4302.00 km^2^. The cropland areas converted from grassland, water, and construction land were 250.59 km^2^, 989.61 km^2^, and 987.47 km^2^, respectively. The areas of the conversion of these three land use types to cropland were relatively low compared to those for forest.

**Figure 3:**
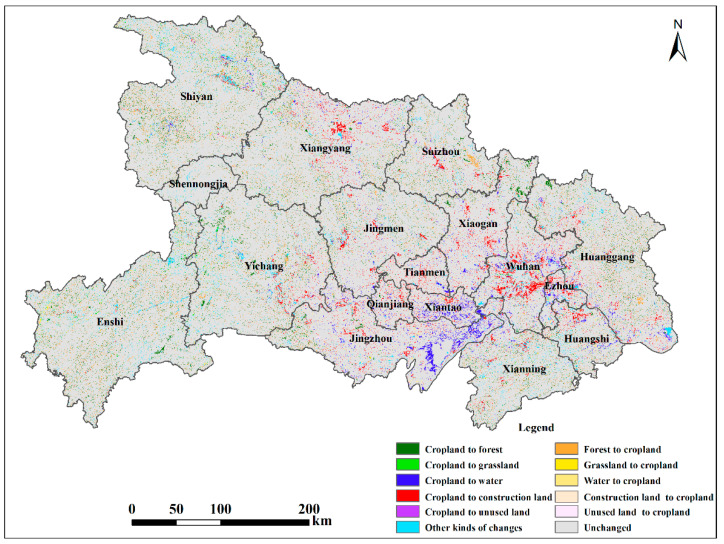
Land use conversion between 2000 and 2018.

**Figure 4:**
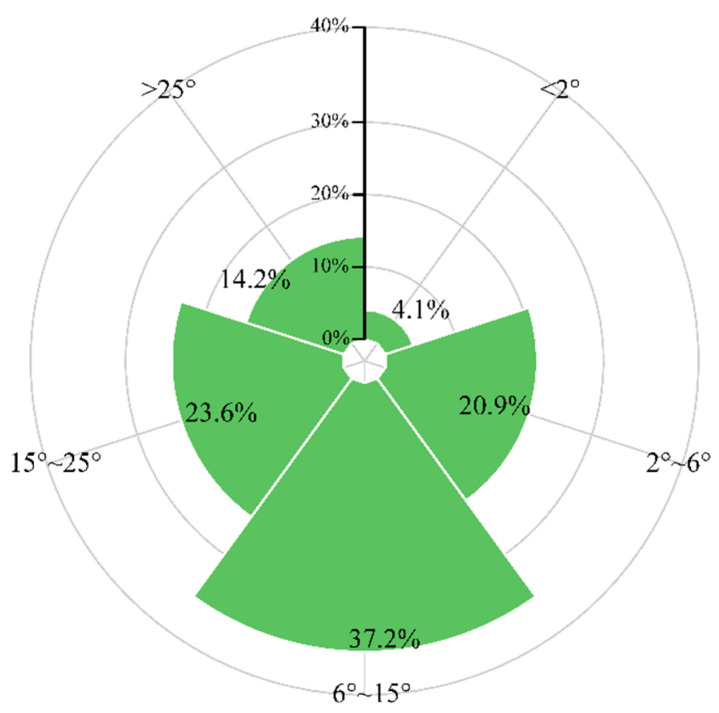
Percentage of the slope grade for cropland conversion to forest and grassland between 2000 and 2018.

The land use transitions were not evenly distributed across Hubei Province or different cities (Figure 3). The increased construction land was mainly located around the original urban areas and large towns, especially in areas of the Jianghan Plain with flatter slopes. Large areas of transition from cropland to forest and grassland were concentrated in Enshi, Shiyan, Yichang, Huanggang, and Xiangyang. According to the statistics of slope grades for the conversion area from cropland to forest and grassland (Figure 4), mountainous and hilly regions with slope grades of 6°–15° and 15°–25° had the greatest proportions, accounting for 37.2% and 23.6% of the total. The increased forest and grassland converted from cropland above 25° accounted for 14.2% of the total.

### 3.2. Variations of Vegetation Coverage

**Figure 5:**
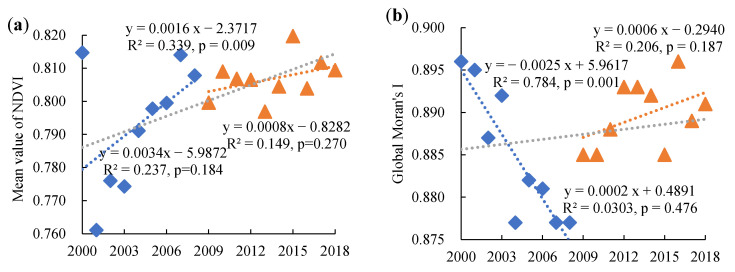
Mean value of NDVI (**a**) and global Moran’s I (**b**) between 2000 and 2018.

The mean NDVI from 2000 to 2018 indicated a significant increasing trend (*p* = 0.009) (Figure 5a). The peak mean NDVI was observed in 2015, with a value of 0.820, while the lowest value occurred in 2001 with a value of 0.761. The change trend was not unanimous, and an obvious upward tendency and a stable fluctuating trend were observed before and after 2008. The overall global Moran’s I of the composite NDVI during 2000–2018 (Figure 5b) exhibited a slight increasing trend (*p* = 0.476), which indicated that the areas of high–high and low–low aggregations of NDVI increased during the period. There was a positive trend for the global Moran’s I after 2008, while the change trend was negative before this time.

**Figure 6:**
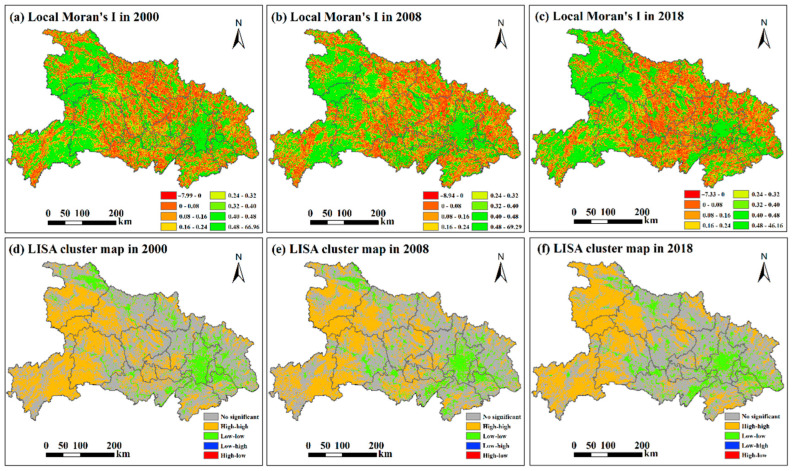
Distribution of local Moran’s I in 2000 (**a**), 2008 (**b**), and 2018 (**c**), and LISA cluster maps in 2000 (**d**), 2008 (**e**), and 2018 (**f**).

As 2008 was a key time point for the vegetation change trend, the local Moran’s I of the vegetation was further analyzed for the years 2000, 2008, and 2018. High positive values of the local Moran’s I were mostly concentrated around Wuhan and in the west of Hubei Province (Figure 6a–c), indicating that the NDVI values in these places were similar to those of their neighbors. The areas with a positive local Moran’s I moved to the northwest in Hubei Province during 2000–2018. Meanwhile, negative local Moran’s I values were mainly observed around the Jianghan Plain and became increasingly concentered in the center of Hubei Province. The LISA analysis maps (Figure 6d–f) show that the areas of the high–high spatial clusters, which represent high NDVI values and high concentrations of high NDVI values, were mainly located in the west of Hubei Province and moved to the northwest between 2000 and 2018. The high–high spatial cluster areas in the Jianghan Plain significantly decreased during this period. Low–low agglomeration areas that had low NDVI values and low concentrations of NDVI values were mainly concentrated in and around Wuhan. Other low–low agglomeration areas occurred in the north of Xiangyang, the northwest and southeast of Jingzhou, and the southeast of Yichang in 2018.

### 3.3. The Change Trend and the Turning Point of the Grain Yield

The change trend of the yield of the five specific farm crops and the total grain yield for Hubei Province and its cities during 2000 and 2018 are listed in Table 3. The grain yield in Hubei Province showed a significant increasing trend at the 0.001 level, and the increasing rate was 44.237 × 10^4^ tons yr^−1^. The annual yield of rice, wheat, and corn also showed significant increasing trends at the 0.001 level, with change rates of 23.983 × 10^4^ tons yr^−1^, 15.172 × 10^4^ tons yr^−1^, and 9.906 × 10^4^ tons yr^−1^, respectively. However, tubers and soybean yields decreased significantly at the 0.05 level.

There were obvious regional variations in the monotonic trends of yields of grain and the five farm crops in the 17 cities during the study period. Grain yield for almost all of the cities showed an increasing trend, except for Wuhan and Enshi. The increasing rates of grain yield for the other 15 cities varied from 0.027 × 10^4^ tons yr^−1^ to 14.748 × 10^4^ tons yr^−1^ with a mean value of 3.4694 × 10^4^ tons yr^−1^, among which the trends were not significant in only three cities, Huangshi, Yichang, and Shennongjia. The rice yield showed a decreasing trend in Wuhan and a significant downward trend at the 0.001 level in Enshi. The other cities had significant increasing trends in rice yield except for Yichang, and the average increasing rate of rice yield was 1.703 × 10^4^ tons yr^−1^. There were only three cities (Huanggang, Enshi, and Shennongjia) and one city (Shennongjia) that had a decreasing trend for wheat yield and corn yield, respectively. The annual tubers yield exhibited significant decreasing trends in Wuhan, Huangshi, Xianning, and Xiantao. A decreasing trend in annual tubers yield also occurred in Yichang, Xiangyang, and Jingzhou, but the change trends were not significant. The average upward and downward change rate in tubers yield were 0.256 × 10^4^ tons yr^−1^ and −0.093 × 10^4^ tons yr^−1^. The annual soybean yield exhibited increasing trends for four cities and showed decreasing trends for the other cities. A significant increasing trend in soybean yield occurred in Enshi (*p* < 0.01) and Shiyan (*p* < 0.05), while for the other cities the decreasing trend was significant except for Ezhou, Jingmen, Suizhou, Qianjiang, and Tianmen.

**Figure 7:**
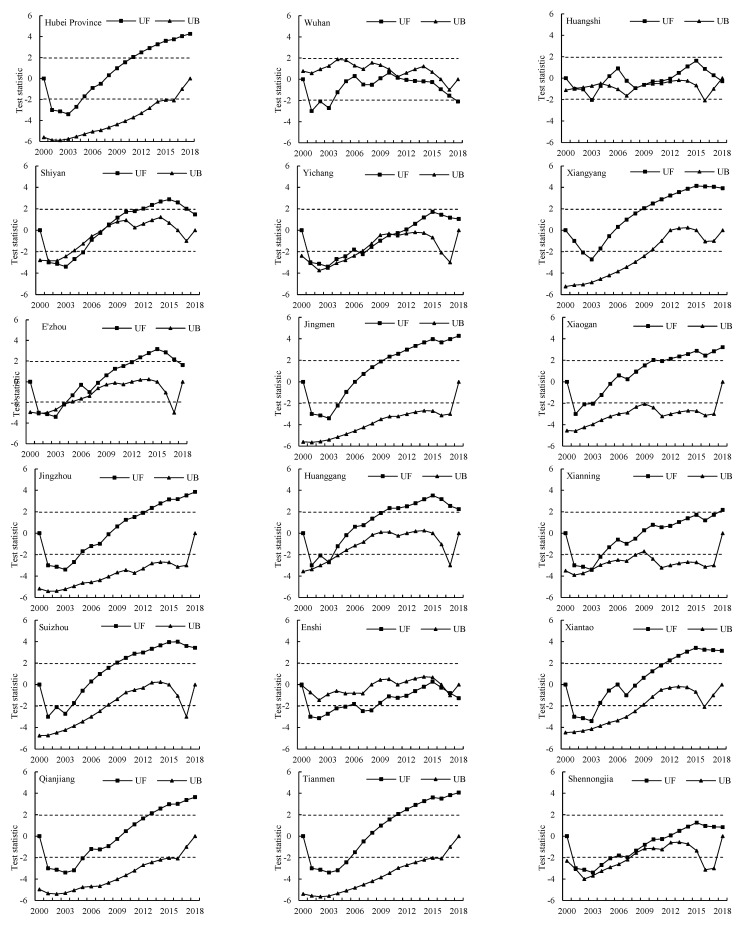
Trend variation and abrupt change test for grain yield between 2000 and 2018. The horizontal dashed lines represent the critical values of the 0.05 significance level.

The trend variation and Mann–Kendall test for abrupt changes in the total grain yield between 2000 and 2018 are shown in Figure 7. The forward curve of Hubei Province showed a decreasing trend from 2000 to 2007 and an increasing trend from 2008 to 2018. Moreover, the downward and upward trends were significant during 2001–2004 and 2011–2018, respectively. Most of the cities had similar change trends in grain yield to that of Hubei Province, including Xiangyang, Ezhou, Jiangmen, Xiaogan, Jingzhou, Huanggang, Suizhou, Xiantao, Qianjiang, and Tianmen. In these cities, decreasing trends in grain yield were detected during the first 6–10 years, among which significant decreasing trends occurred for the first 4–6 years. Additionally, grain yield in these cities indicated an increasing trend during the last 9–13 years, and significant increasing trends occurred during the last 6–10 years. There were several cities that had different characteristics to their change trends. The grain yield of Shiyan indicated a decreasing trend from 2000 to 2007, and the opposite trend was observed from 2008 to 2018. There was a significant decrease and increase trend during 2001–2005 and 2012–2017, respectively, and the values of the forward curve exceeded the critical value lines. A turning point in grain yield for Shiyan was identified in 2008 at the 0.05 significance level with the point of intersection for the forward curve and the backward curve indicating that an abrupt change occurred at that time. The grain yield for Yichang, Xianning, and Shennongjia had similar change trends to Shiyan, on the whole. There was a decreasing tendency during the first 9 (Xianning) or 12 (Yichang and Shennongjia) years, which was significant during 2001 to 2004 (Xianning) and 2001 to 2007 (Yichang and Shennongjia). For the other periods, the grain yield for these three cities showed slight increasing trends. Meanwhile, for Yichang, the forward and the backward trend of grain yield intersected in 2010, which indicated a turning point. There was a significant decreasing trend for Wuhan before 2004 and for Huangshi before 2003. The fluctuating range was relatively stable for these two cities, and almost all the values of the forward and backward curves were within the critical value lines during this period.

## 4. Discussion

### 4.1. Ecological Restoration Conditions

The increasing trend of the mean NDVI during 2000–2018 indicated that the vegetation recovered gradually, as a whole. This change trend was attributed to various factors. The climate has exhibited an increasing trend in recent decades, which could extend the length of the growing season and intensify productivity [53]. Though climate boosts vegetation growth, the effects of anthropogenic factors cannot be ignored [54]. The large percentage of land use conversion from cropland to forest or grassland could have resulted in vegetation greening [55]. Ecological restoration, and especially the returning farmland to forests project, has proven to be one of the main driving variables for vegetation restoration [49,56].

The decrease in the global Moran’s I before 2008 may be attributed to the relatively low vegetation coverage of the areas converted from cropland to grassland or forest in the early stages. With an increase in the vegetation coverage, the global Moran’s I increased beginning in 2008 and maintained a stable fluctuating trend from 2012 to 2018. The high–high agglomeration was mainly distributed in the west of the study area, which comprised mountainous areas with relatively steep slopes and high elevations. These places had superior vegetation resources due to a specific topography and suitable climate conditions, as well as low population density. The increasing concentration of the NDVI in the west and northwest of the study area was probably a result of the high ratios of steeply sloped cropland, which has been gradually restored to forest and grassland since the implementation of the policy. Moreover, the increasing concentration of the NDVI in these places may also be attributed to the protection of natural resources in the past. The growth of vegetation was relatively high in these superior natural resource endowments with high vegetation coverage [34], making the high–high areas more concentrated, especially in the northwest. The decrease in the high–high areas in the Jianghan Plain is mainly attributed to the flat terrain and the development of the economy. The landscapes were fragmented, and rapid urban expansion resulted in a decrease in the spatial autocorrelation of NDVI. Although vegetation has been restored in some steeply sloped cropland since the implementation of the returning farmland to forests project, high–high spatial autocorrelation did not increase very much due to landscape fragmentation and economic development. The decline in the low–low NDVI area in Wuhan indicates that the vegetation coverage gradually increased, especially in the northern and southern parts.

### 4.2. Effects of the Returning Farmland to Forests Project on Grain Production

The overall area of cropland of Hubei Province exhibited a decreasing trend from 2000 to 2018; however, the result of the Mann–Kendall trend analysis on the grain yield indicated a significant increase in this period. Thus, the grain production of Hubei Province was not threatened by the returning farmland to forests project as a whole. This is attributed to the comprehensive influences of the driving factors on grain production.

During the first 5–11 years following the implementation of the policy, there was a downward trend in the grain yield in most cities in Hubei Province, indicating that grain yield was affected by this project to some degree. The decrease in grain yield had a duration of approximately 11 or more years in Yichang, Shennongjia, and Enshi, which had steep slopes and high elevations (Table 4). Meanwhile, in cities with flat and gentle slopes, the duration of the downward trend in grain yield was much shorter. As the aim of the returning farmland to forests project was to reduce soil erosion by shifting cropland with steep slopes into forest or grassland, steeper cultivated land was more likely to be affected, which further reduced grain production. Urbanization was another significant reason for the decrease in the grain yield. Large areas of cropland were converted to construction land (Table 2), which were mostly flat and had good productivity [57]. At the beginning of the 21st century, the phenomenon of rural-to-urban migration occurred, and large numbers of framers swarmed into cities, resulting in the abandoning of farmland and a decrease in grain yield [27].

**Figure 8:**
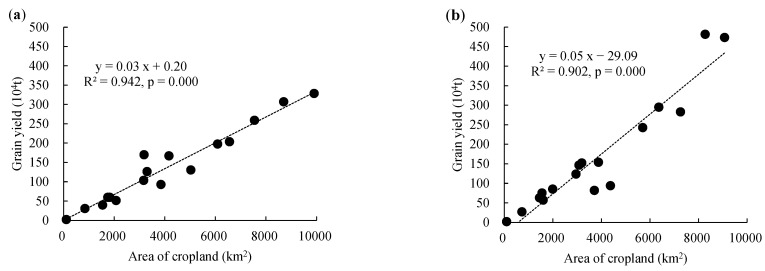
The relationship between grain yield and cropland area in different cities in 2000 (**a**) and 2018 (**b**).

With the exception of a few cities (such as Wuhan, Huangshi, Enshi, and Shennongjia), grain yield exhibited a significant upward trend in most regions of Hubei Province during the last 6–10 years. Large areas of cropland that had steep slopes were converted to forest and grassland due to the returning farmland to forests project. Additionally, some cropland was replaced by construction land due to the development of society and urbanization, which affected grain yield to some degree. However, the newly-added cropland converted from forest and other kinds of land use types partly promoted an increase in the grain yield. Per unit area yield is a dominant factor for grain production. The most effective method to simultaneously maintain grain production and ecological integrity is to increase the potential productivity or the per unit area yield of the existing cropland [58]. The relationships between grain yield and cropland area for the cities in 2000 and 2018 are illustrated in Figure 8. The fitting curve in 2018 had a steeper slope, indicating that the overall productivity of the cropland increased, which can be attributed to technological progress in terms of chemical fertilizers, pesticides, crop seeds, etc. Thus, the total grain yield was not influenced by the decrease in the cropland area as a whole. Furthermore, in order to alleviate cropland loss, China has implemented strict cropland protection policies to ensure grain production, including the “Permanent Basic Farmland Protection”, “Farmland Occupation Compensation System”, and “High-Standard Basic Farmland Construction” policies, which have achieved great results in terms of increasing grain yield [30].

### 4.3. Comparison with Other Studies

Some previous studies have focused on the responses of vegetation restoration conditions and grain production to the returning farmland to forests project. The methods and perspectives of this study differ from those of related studies. The spatial autocorrelation of the vegetation and the change trend of the grain yield were explored based on Moran’s I and the Mann–Kendall test. The results of this study show that there has been a significant restoration of vegetation since the implementation of the project and that the vegetation change also had some regional variations. In this regard, they are similar to the results of previous studies [49,59]. Food security is always a worldwide concern due to its fundamental importance for the survival of human beings [60]. Most countries have expended great effort to increase their grain production in order to safeguard their food supply [61]. Apart from water shortages, soil pollution, and climate change, cropland resource is a key element of grain production [60,62]. Much attention has been paid to the relationships between cropland resources and grain production in other studies. In the context of urban–rural transformation and development, a significant grain production transition can be detected in the process of farmland transition in China, and the characteristics of farmland transition could help to regulate grain production transition effectively [63]. The results of Liu et al. [64] indicated that cropland loss due to population increase had a negative influence on grain production. With respect to grain production, the results of this study and those of previous studies [65,66] indicate that grain yield is affected in the early stages following the implementation of a project; however, a series of positive factors mean that the grain yield subsequently increases. This study focused on the regional change trend in grain production using the Mann–Kendall test, which can detect the potential breakpoint of the grain yield. The results of this study can help policymakers to devise more efficient measures or policies based on regional characteristics to achieve a balance between ecological restoration and grain production.

### 4.4. Enlightenment and Suggestions

The total grain yield in Hubei Province and most of its cities decreased during the early stages following the implementation of the policy of returning farmland to forests. Large areas of cropland with steep slopes were transformed to forests or grassland, which resulted in a direct decrease in the total grain yield. However, grain production was not threatened due to the influence of some positive factors. First of all, cropland decrease was alleviated due to the newly added cropland converted from forest and other land use types. Moreover, cropland with steep slopes converted to forest or grassland is more environmentally friendly and has less of an impact on grain production [57]. The results of this study also show that the productivity of cropland increased during the study period, indicating that more grain output could be generated with the same cropland area. Since the total grain output has been continuously maintained in recent years in central China, the Loess Plateau [66], and China as a whole [37], the policy of returning farmland to forests should be continued in order to alleviate soil erosion and restore the ecological environment.

As grain production still faces significant challenges for a variety of reasons [67], effective measures should also be taken to ensure grain production. It has been confirmed that not all cropland with a steep slope is exposed to severe soil erosion, so choosing the cropland that needs to be restored should be considered as well as the soil erosion conditions of the cropland [57]. This can help to maintain both the cropland area and grain output, to some degree. The government should improve and enhance the current cropland protection system and the grain production system by increasing investment in basic cropland infrastructure, protecting neo-cultivated cropland from degradation [37], and so on. Economic measures, such as improving financial subsides, should be taken to ensure the protection of basic croplands [68]. Cropland consolidation can improve cropland productivity potential and thus should be adopted in suitable regions. Scientific research into improving the per unit area yield and the selection and breeding of fine varieties of different farm crops should be encouraged to promote an increase in grain production.

## 5. Conclusions

This study explored the vegetation dynamics and food security of Hubei Province against the background of ecological restoration. Spatial autocorrelation and the Mann–Kendall test were applied to examine the agglomeration of NDVI and the change trend of grain yield. The results showed that vegetation coverage was restored gradually and that there has been an increase in the mean NDVI since the project of returning farmland to forests was implemented. However, the spatial agglomeration of NDVI exhibited a downward trend from 2000 to 2008, and an upward trend was observed from 2009 to 2018. High–high agglomeration was mainly found in the mountainous areas in the western part of the study area and gradually moved to the northwest, while the high–high areas in the Jianghan Plain decreased during the study period. Though the total cropland area decreased from 2000 to 2018, the grain yield in Hubei Province and most of its cities exhibited a significant upward trend as a whole, indicating that food security was not threatened by the implementation of the returning farmland to forests project. During the first 5–11 years, the grain yield showed a downward tendency for Hubei Province and almost all cities, which may be attributed to the sharp decrease in the cropland area, resulting from the conversion of cropland to forests or grassland. The grain yield was more sensitive in cities with steep slopes, and the duration of the grain yield decrease was longer in these areas. Meanwhile, during the last 6–10 years, an increasing trend was observed in terms of grain yield in most cities, which can primarily be attributed to the newly-added cropland converted from other kinds of land uses and an increase in cropland productivity. Thus, as an efficient ecological restoration practice, the returning farmland to forests project should continue to be implemented in order to eliminate soil erosion and promote environmental quality. The methods used in this study could also be applied in some related research into vegetation and grain production dynamics. In addition, effective measures, including strict cropland protection policies, financial measures, and scientific research into improving cropland productivity, should be taken or enhanced to promote an increase in grain yield.

## Figures and Tables

**Table 1:** Statistics of the areas (km^2^) and ratios (%) of the land use types in Hubei Province in 2000 and 2018.

Land Use Types	2000		2018		Change (2018–2000)	
	Area	Ratio	Area	Ratio	Area	Ratio
Cropland	69,650.07	37.47	65,355.20	35.16	−4294.87	−6.17
Forest	92,782.39	49.92	92,259.35	49.64	−523.03	−0.56
Grassland	7044.34	3.79	6883.94	3.70	−160.40	−2.28
Water	10,809.80	5.82	12,398.25	6.67	1588.44	14.69
Construction land	5145.86	2.77	8604.11	4.63	3458.25	67.20
Unused land	436.53	0.23	368.15	0.20	−68.38	−15.67

**Table 2:** The conversion matrix of different land uses between 2000 and 2018 (km^2^).

Land Use Types	Cropland	Forest	Grassland	Water	Construction Land	Unused Land
Cropland	58,786.92	4676.43	285.61	2381.35	3480.68	39.09
Forest	4302.00	86,359.84	754.36	555.47	805.04	5.67
Grassland	250.59	859.28	5800.61	65.58	64.80	3.48
Water	989.61	262.54	33.19	9168.55	285.15	70.76
Construction land	987.47	93.85	7.36	99.75	3953.77	3.66
Unused land	38.61	7.41	2.81	127.54	14.68	245.48

**Table 3:** Mann–Kendall trend analysis for the total grain yield and the yield of five specific farm crops from 2000 to 2018.

Region	Grain		Rice		Wheat		Corn		Tubers		Soybean	
	Z	β (10 ^4^ Tons yr ^−1^)	Z	β (10 ^4^ Tons yr ^−1^)	Z	β (10^4^ Tons yr ^−1^)	Z	β (10 ^4^ Tons yr ^−1^)	Z	β (10 ^4^ Tons yr ^−1^)	Z	β (10 ^4^ Tons yr ^−1^)
Hubei	4.898 ***	44.237	4.968 ***	23.983	4.478 ***	15.172	4.688 ***	9.906	−2.309 *	−2.785	−2.379 *	−0.993
Wuhan	−1.399	−0.696	−1.609	−0.721	0.770	0.055	2.449 *	0.230	−2.239 *	−0.119	−4.128 ***	−0.139
Huangshi	0.385	0.080	3.359 ***	0.439	2.519 *	0.119	0.735	0.026	−4.446 ***	−0.288	−2.382 *	−0.054
Shiyan	2.099 *	1.770	2.099 *	0.309	1.749	0.310	2.415 *	0.814	0.910ns	0.247	2.309 *	0.101
Yichang	1.679	0.800	0.210	0.060	3.569 ***	0.430	4.058 ***	0.878	−0.070	−0.022	−2.836 **	−0.065
Xiangyang	4.548 ***	14.748	2.589 **	1.973	4.618 ***	8.766	4.968 ***	4.678	−0.035	−0.025	−3.886 ***	−0.146
Ezhou	2.239 *	0.458	2.519 *	0.419	1.190	0.025	0.210	0.002	0.910	0.058	−1.297	−0.009
Jingmen	4.898 ***	6.444	4.268 ***	3.552	3.289 **	1.735	4.828 ***	0.985	0.700	0.092	−0.631	−0.014
Xiaogan	3.848 ***	3.610	3.149 **	2.850	1.749	0.525	4.058 ***	0.188	1.085	0.203	−3.569 ***	−0.153
Jingzhou	4.478 ***	9.086	3.988 ***	6.319	5.038 ***	2.416	0.770	0.203	−0.875	−0.030	0.910	0.090
Huanggang	2.869 **	4.524	2.869 **	3.084	−0.980	−0.170	3.359 ***	0.312	1.889	1.106	−4.446 ***	−0.178
Xianning	2.799 **	1.355	3.149 **	1.345	3.610 ***	0.083	1.329	0.044	−2.869 **	−0.140	−2.379 *	−0.067
Suizhou	4.058 ***	3.140	3.429 ***	1.761	3.429 ***	1.231	2.799 **	0.314	1.051	0.313	−1.614	−0.026
Enshi	−0.560	−0.176	−2.659 ***	−0.363	−5.356 ***	−0.234	3.778 ***	0.882	0.700	0.320	2.659 **	0.070
Xiantao	3.778 ***	1.566	3.219 **	0.880	4.376 ***	0.533	3.079 **	0.295	−2.065 *	−0.025	−2.029 *	−0.029
Qianjiang	4.268 ***	1.772	3.429 ***	1.095	3.816 ***	0.428	1.399	0.075	0.630	0.012	−0.385	−0.005
Tianmen	4.688 ***	2.661	4.516 ***	1.458	4.548 ***	0.760	2.239 *	0.065	1.609	0.075	0.000	−0.001
Shennongjia	1.541	0.027	2.043 *	0.000	−1.165	0.000	−1.681	−0.011	3.009 **	0.030	1.355	0.001

*** Indicates significant at the 0.001 level. ** Indicates significant at the 0.01 level. * Indicates significant at the 0.05 level.

**Table 4:** Statistics on the elevation and slope in the study area.

Region	Elevation (m)		Slope (°)	
	Mean	Standard Deviation	Mean	Standard Deviation
Hubei Province	431.6	494.2	13.6	11.3
Wuhan	37.7	46.2	5.7	4.9
Huangshi	109.5	127.1	10.7	9.3
Shiyan	736.9	387.8	21.6	10.8
Yichang	662.3	508.2	18.8	12.0
Xiangyang	347.5	358.1	12.5	10.3
Ezhou	31.4	31.9	5.3	4.9
Jingmen	116.5	104.7	8.5	6.8
Xiaogan	75.2	83.8	6.6	6.0
Jingzhou	42.6	57.8	5.5	4.8
Huanggang	173.8	197.6	10.3	8.2
Xianning	184.9	196.8	12.1	9.8
Suizhou	185.6	127.2	9.4	7.2
Enshi	1074.9	356.4	21.1	11.5
Xiantao	26.3	5.9	3.7	2.8
Qianjiang	28.1	8.4	5.3	4.2
Tianmen	31.3	8.5	4.2	3.5
Shennongjia	1683.4	469.4	27.3	11.3

## Data Availability

Data and materials are available from the corresponding authors upon request.
